# Metastatic Basal Cell Carcinoma Accompanying Gorlin Syndrome

**DOI:** 10.1155/2014/362932

**Published:** 2014-11-19

**Authors:** Yeliz Bilir, Erkan Gokce, Banu Ozturk, Faik Alev Deresoy, Ruken Yuksekkaya, Emel Yaman

**Affiliations:** ^1^Department of Internal Diseases, Faculty of Medicine, Gaziosmanpaşa University, 60100 Tokat, Turkey; ^2^Department of Radiodiagnostics, Faculty of Medicine, Gaziosmanpaşa University, 60100 Tokat, Turkey; ^3^Division of Medical Oncology, Faculty of Medicine, Gaziosmanpaşa University, 60100 Tokat, Turkey; ^4^Department of Pathology, Faculty of Medicine, Gaziosmanpaşa University, 60100 Tokat, Turkey; ^5^Department of Medical Oncology, Faculty of Medicine, Mersin University, 33343 Mersin, Turkey

## Abstract

Gorlin-Goltz syndrome or basal cell nevus syndrome is an autosomal dominant syndrome characterized by skeletal anomalies, numerous cysts observed in the jaw, and multiple basal cell carcinoma of the skin, which may be accompanied by falx cerebri calcification. Basal cell carcinoma is the most commonly skin tumor with slow clinical course and low metastatic potential. Its concomitance with Gorlin syndrome, resulting from a mutation in a tumor suppressor gene, may substantially change morbidity and mortality. A 66-year-old male patient with a history of recurrent basal cell carcinoma was presented with exophthalmus in the left eye and the lesions localized in the left lateral orbita and left zygomatic area. His physical examination revealed hearing loss, gapped teeth, highly arched palate, and frontal prominence. Left orbital mass, cystic masses at frontal and ethmoidal sinuses, and multiple pulmonary nodules were detected at CT scans. Basal cell carcinoma was diagnosed from biopsy of ethmoid sinus. Based on the clinical and typical radiological characteristics (falx cerebri calcification, bifid costa, and odontogenic cysts), the patient was diagnosed with metastatic skin basal cell carcinoma accompanied by Gorlin syndrome. Our case is a basal cell carcinoma with aggressive course accompanying a rarely seen syndrome.

## 1. Introduction

Gorlin syndrome, one of the hereditary syndromes concomitant with multiple basal cell carcinoma (BCC), is associated with many anomalies observed in several systems [1]. This autosomal dominant syndrome is characterized by multiple BCC seen in the early stage of the life, palmar and plantar pits, mandibular prognathism, mandibular keratocysts, hypertelorism, falx cerebri calcification, spina bifida, bifid costa, vertebral abnormalities, ovarian fibroma, cardiac fibromas, milia, epidermal cysts, mesenteric cysts, gastric polyps, medulloblastoma, meningioma, and fatal rhabdomyoma [1]. Odontogenic keratocysts are characteristic in this syndrome. PTCH (Drosophila patched gen human analogue) is localized on chromosome 9q22.3-q31 and encodes sonic hedgehog (SHH) signaling pathway. Autosomal dominant germline mutations on this gene account for the pathogenesis of Gorlin syndrome. The inactivation of PTCH gene causes cancer formation, mainly by leading to overexpression in the SHH pathway and basal cell carcinoma [2].

Basal cell carcinoma (BCC) is the most commonly seen skin cancer, which accounts for 80% of the nonmelanoma skin cancers [3]. BCC is most commonly seen between the ages of 40 and 80 years old, with the incidence increasing over time. Lifetime risk for BCC was reported to be 1/18 in men and 1/20 in women. Environmental and hereditary factors have a substantial contribution to the development of BCC. In its etiology, most likely causal factor is ultraviolet-B (UVB) beams. Clear skin color and prolonged sun exposure are important risk factors. Tumor may show a course with local recurrences, but its potential for metastasis is low [4, 5]. The risk for metastasis occurrence is 0.0028–0.55% [6]. Majority of the metastases originate from facial BCCs. Most commonly seen metastatic sites include lymph node (60%), lungs (42%), bone (20%), and skin (10%) [7]. Incidence of pulmonary metastasis is approximately 0.1% [8].

Gorlin syndrome is a syndrome with a high risk for malignity development at earlier ages, which may be diagnosed during the childhood based on physical findings. Our case is a basal cell carcinoma with diffuse lung metastases, which has a course with recurrent multiple basal cell carcinoma and which is accompanied by Gorlin syndrome diagnosed at advanced age.

## 2. Case Report

**Figure 1:**
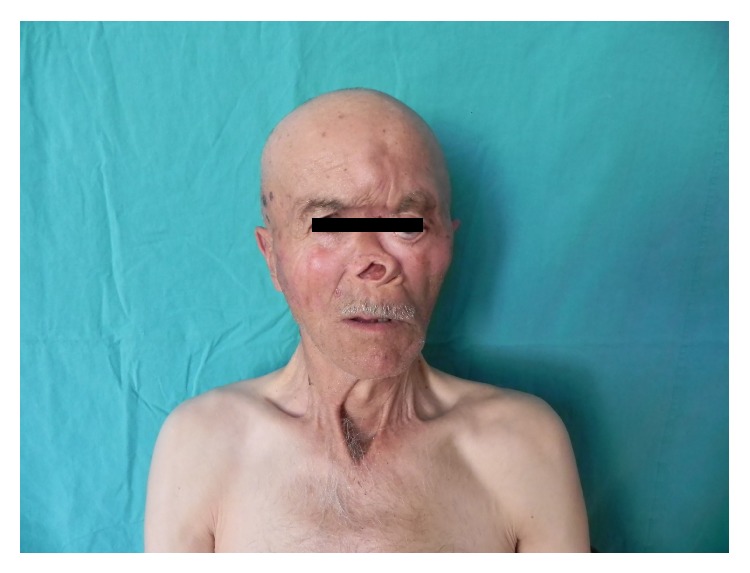
The facial profile of the patient.

The medical history of the 66-year-old male patient, who has been presented to the hospital with exophthalmus and skin lesions, revealed that he had firstly undergone local excision due to basal cell skin carcinoma located on his face 12 years ago, that new lesions had formed 2 cm below the left oculonasal junction and in the left nasal root 6 years following the first diagnosis, and that the mass has been diagnosed to be a recurrence of BCC that indented medial wall of the left orbita and that expanded to close bones. The patient had undergone rhinectomy and reconstructive surgery. He had received radiotherapy. Six years after the completion of the therapy, he was presented with a lesion near the left eye (Figure 1).

**Figure 2:**
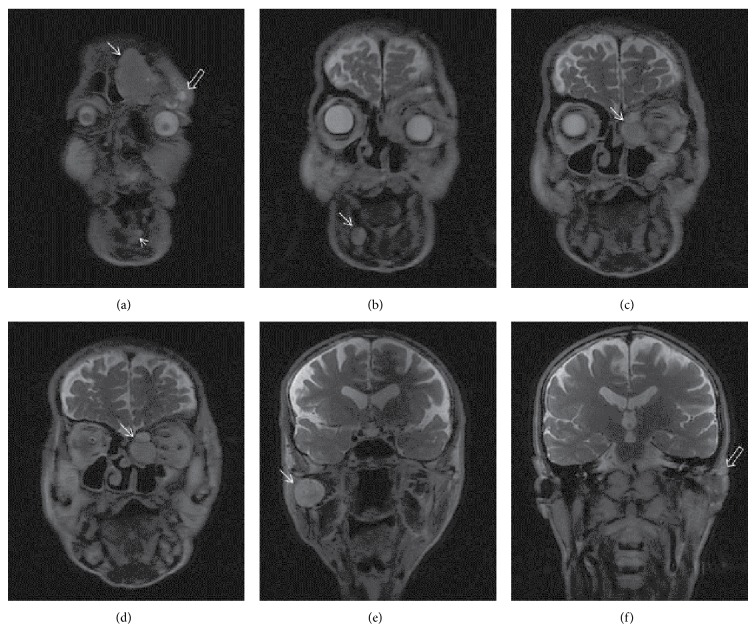
Coronal T2-weighed MR imaging: (a) a lobular hypodense mass caused destruction and expansion at frontal sinus like a mucosel or keratocyst (arrow mark). Cystic mass at left orbital wall and mandibular corpus (arrow marked). (b) Dens odontogenic keratocysts at right mandibular corpus (arrow mark). (c), (d) Multiple cystic lesions with different size and dens at left ethmoidal sinuses (marked arrow). (e) A round, dense cystic lesion with expansil regular contour was observed in the right mandibular ramus. (f) A soft tissue mass with heterogeneous and irregular contour, which contained cystic-necrotic areas starting from left auricula, extended to external ear tract (arrow mark).

Facial MRI of the patient revealed a dense and large cystic mass lesion that led to an expansion of left frontal sinus by filling it and that destructed the anterior wall (Figure 2(a)). There were ovoid, dense cystic lesions with uniform contours in the central part and round, millimetric cystic lesions in the right of the mandibular corpus. A cystic lesion with uniform contours was observed in the contiguity of the retromolar dental roots in the left maxillar bone (Figures 2(a) and 2(b)).

**Figure 3:**
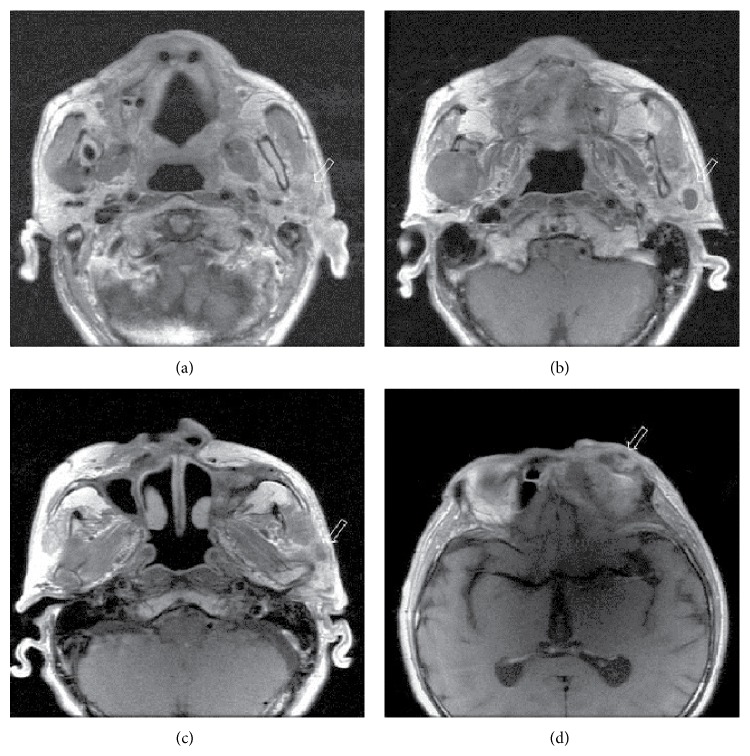
Contrast enhanced axial T1-weighed MR imaging: (a), (b), and (c) a heterogeneous soft tissue mass with cystic-necrotic components at left parotid region with extending auricula and external ear tract (light arrow mark). (d) A heterogeneous soft tissue mass with cystic-necrotic components at orbital roof with destruction of frontal bone (light arrow mark).

**Figure 4:**
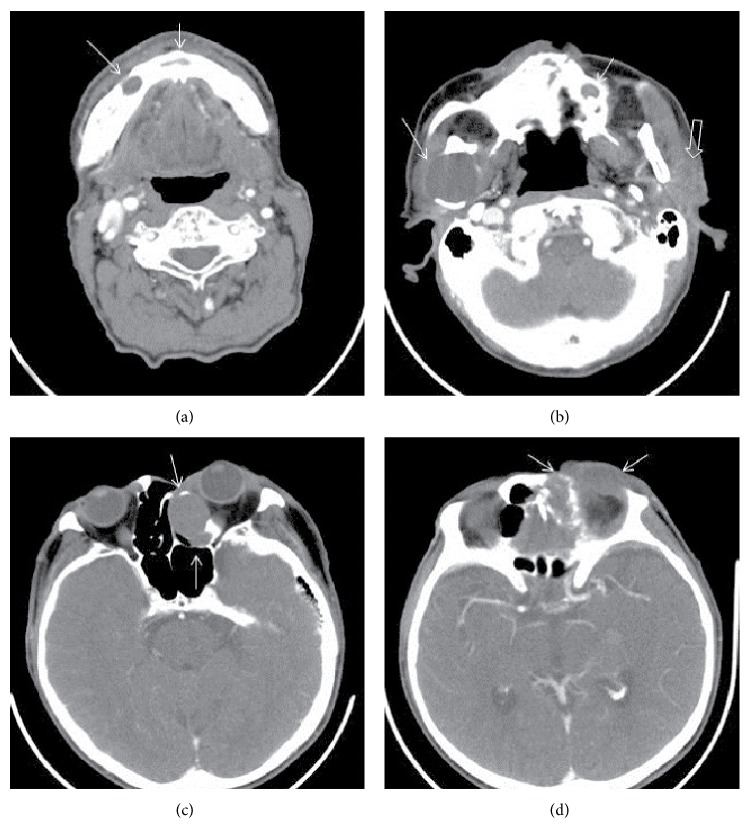
Contrast enhanced CT images of neck: (a) cystic mass lesions with regular margins at mandibular corpus (arrow marked). (b) A round cystic lesion with lytic expansil regular contour at right ramus mandible and small cystic lesion at maxillar bone (marked arrow), a heterogeneous contrasted mass at left parotid region (light arrow marked). (c) Two lytic expansil cystic lesions at left ethmoidal cells. (d) A heterogeneous contrasted mass with irregular margins at subcutaneus tissue of left orbital wall with destruction of frontal bone.

In the left, there were 2 contiguous cystic lesions with different sizes and contents and with regular contours in the ethmoid air cells, showing indentation to left medial orbita, with a expansile-destructive characteristic (Figures 2(c) and 2(d)). A round, dense cystic lesion with expansil regular contour was observed in the right ramus mandibula (Figure 2(e)). In the hard palate, clefting and morphology consistent with highly arched palate were observed (Figures 2(a)–2(d)). There was a soft tissue mass with heterogeneous and irregular contour, which contained cystic-necrotic areas inside and which, starting from left auricula, extended to external ear tract and to parotid lodge. Another lesion with similar appearance was localized in the orbital roof (Figure 2). In the contrasted series, we observed a soft tissue mass with heterogeneous contrast and irregular contour with cystic-necrotic areas inside, which, starting from left auricula, extended to external ear tract and to parotid lodge (Figures 3(a), 3(b), and 3(c)). Similarly, on the left orbital roof, there was a soft tissue mass that led to destruction in the inferior part of the frontal bone (Figure 3(d)). Contrasted neck CT revealed soft tissue lesions and cystic masses that formed expansion-destruction in the cranial bone structures (Figure 4). Odontogenic keratocysts that were described suggested the presence of Gorlin syndrome in the patient.

**Figure 5:**
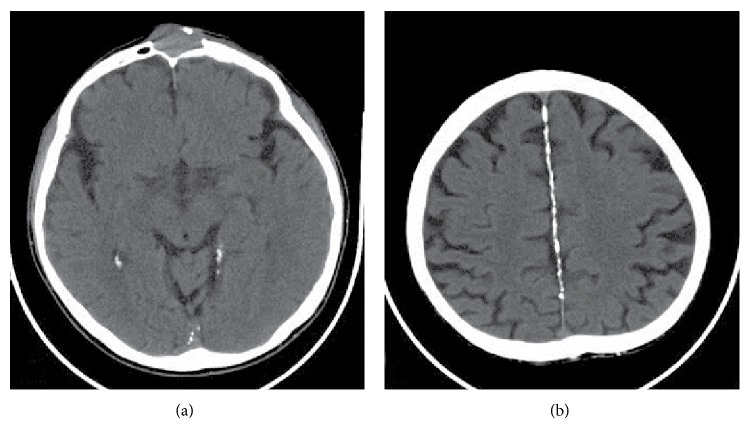
CT imaging of brain: (a) calcifications at tentorium and mass at frontal sinus with bone destruction and expansion. (b) Diffuse calcification at falx cerebri.

**Figure 6:**
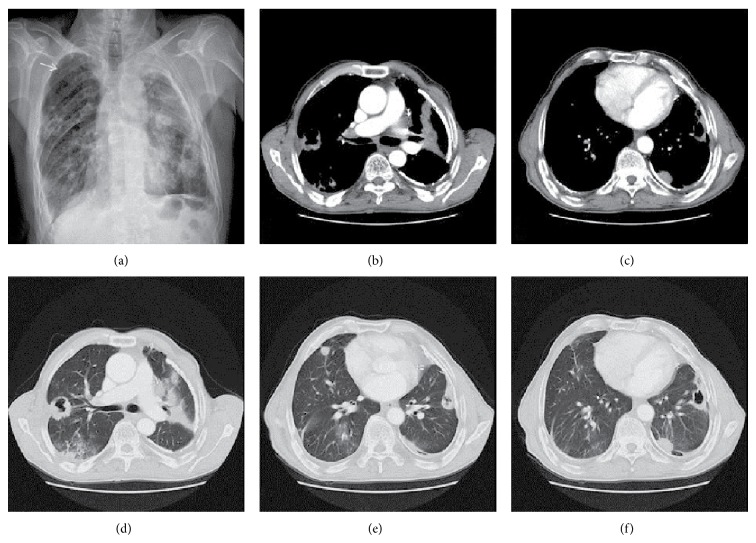
(a) X-ray imaging of lung. Bifid costa, multiple irregular cavity, and metastatic nodules at the lungs. (b)–(f) CT imaging of thorax: (b) irregular cavitary and nodular metastatic lesions at right upper pulmonary segments and pleural calcifications. (c) Left pulmonary irregular cavitary and nodular metastatic lesions. (d), (e), and (f) Multiple solid and cavitary metastatic pulmonary nodules.

Other findings of Gorlin syndrome were investigated. The patient had hearing loss, gapped teeth, highly arched palate, and frontal prominence. Brain CT of the patient showed diffuse lamellar-slightly modular calcifications in the falx cerebri and focal calcifications in the tentorium (Figure 5). Lung X-ray showed bifid costa anomaly in the right 1st costa. In both lungs, there were the opacities for the lesions suggesting nodular metastasis with irregular thick cavitary appearance and irregular contours. In the left apex, opacity was increased and rough calcifications were present on the pleural surfaces (Figure 6(a)). Due to clinical and radiological findings that accompanied BCC lesions, Gorlin-Goltz syndrome was considered.

Thoracic CT performed upon the observation of nodules suggesting the metastasis on the lung X-ray revealed very numerous cavitary masses with irregular contours and thick walls and solid metastatic mass lesions of various sizes with irregular contours that showed parenchymal spiculated extensions in both lungs (Figures 6(b)–6(f)).

**Figure 7:**
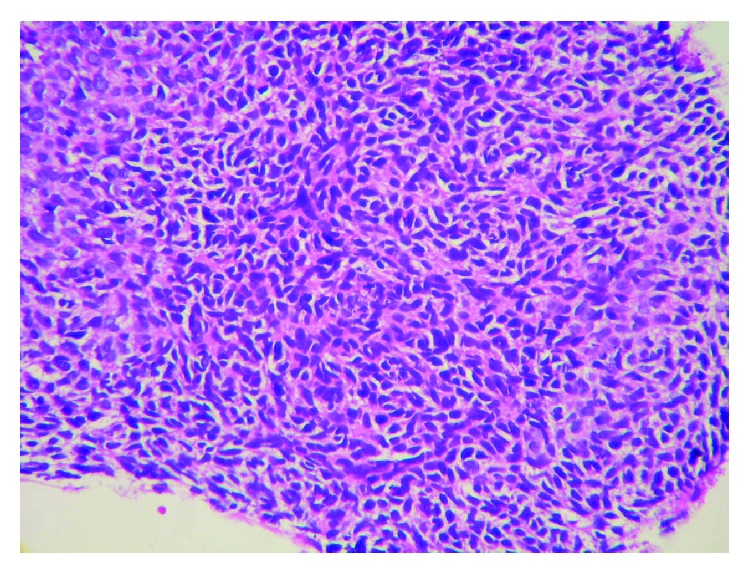
In the patient's first biopsy specimen, tumor cell proliferation areas which consist of basaloid cells are seen.

**Figure 8:**
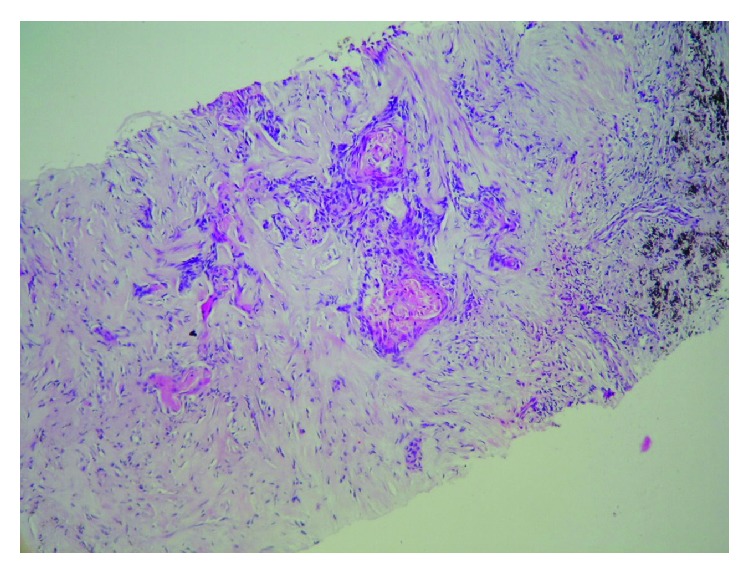
The core biopsy specimen taken from thorax. The neoplastic cell infiltration, which is characterized as admixture of basaloid cells and keratin producing squamous cells, is seen.

The first biopsy sample obtained from the mass located in the ethmoid sinus of the patient was examined (Figure 7). The first biopsy specimen showed a basaloid tumor cell proliferation, which was characterized as basaloid cells with relatively small, round/oval hyperchromatic nuclei and narrow cytoplasms. In some areas peripheral palisading nuclei and retraction artifacts were present, which support basal cell carcinoma diagnosis. Squamoid differentiation or keratin production findings were not seen in any area. Besides the next biopsy specimen which was taken from thorax, the basaloid cell proliferation is admixed with keratin producing squamous cells and has an infiltrative pattern, which was similar to basosquamous carcinoma (Figure 8).

In the light of existing clinical and laboratory findings, the patient was diagnosed with metastatic BCC accompanied by Gorlin syndrome.

## 3. Discussion

In 1996, Gorlin and Goltz reported in two women a syndrome characterized with multiple basal cell carcinoma, the cysts in the chin and bifid costa, which they have called after their name [1]. Gorlin-Goltz syndrome is also called as basal cell nevus syndrome, nevoid basal cell carcinoma syndrome, and Ward syndrome [9]. Despite its autosomal dominant trait, the familial history of the syndrome is negative in 1/3 cases [9].

BCC accounts for 80% of all skin cancers [3]. This tumor originates from pluripotential cells located in the basal layer of the epidermis and in the outer sheath of the hair follicle. Although it is seen in any area with the skin, 85% of the cases are seen in head-neck area. Cumulative exposure to ultraviolet (UV) is known as the main causal factor in the development of basal cell carcinoma. UV was found to cause the development of basal cell carcinoma by leading to mutations in p53 and PTCH tumor suppressor genes [10]. Furthermore, etiological factors of the BCC development include having a light colour of hair, eyes, and skin, sunburn during the childhood and adolescence, ionized radiation, human papilloma virus infections, exposure to inorganic arsenic, infrared radiation, scar tissue, tattoo, sebaceous nevus, trauma, chronic ulcers, and immunosuppression. Basal cell carcinomas of the skin are commonly seen and slowly growing tumours with rarely seen metastases.

Our patient is a case of basal cell carcinoma who had the diagnosis 12 years ago and who underwent 2 resections for local recurrences during that 12-year period. He had UV exposure due to his occupation. In the systemic scans performed at the time of the last recurrence, the lesions primarily suggested the presence of metastasis in bilateral lungs. In the literature, there are limited numbers of case reports with basal cell carcinoma that did systemic metastases and that accompanied to Gorlin syndrome. Fernández-Aceñero et al. reported a rare case with clear cell BCC with Gorlin syndrome and lung metastasis and it was emphasized that the recurrence and metastatic potential are higher in such patients [11].

In the patients with Gorlin syndrome, there was a tendency of development multiple skin cancer with more aggressive clinical course characterised with local and systemic recurrences. Our patient has been followed up with recurrent localized disease for 12 years and, lastly, he had been presented to our clinic local recurrence with cranial bone and sinus invasions and with multiple distant metastases in the lung. There were similar case reports such as lymph node and bone metastasis of BCC in the patients with Gorlin syndrome older than 55 years [12, 13].

Because of aggressive nature of recurrent basal cell cancers in the pts with Gorlin syndrome, chemoprevention will be more important in the future. Like our patient, surgical excision of multiple and extensive skin lesions can cause pain and facial disfigurement. Although several surgical procedures were performed to our patient, the lesions progressed and distant metastases occurred. Close monitoring of skin lesions and avoiding sun exposure are essential for prevention of skin cancer. However our patient was not in compliance with follow-up and sun exposure was still continued. There are some chemopreventive strategies in the literature. Tazarotene, a topical retinoid, was tested for basal cell cancer chemoprevention for 3 years; only 6% pts had a chemopreventive response [14]. Photodynamic therapy (PDT) using methyl aminolevulinate PDT with red light (630 nm) or 20% 5-aminolevulinic acid and blue light is reported as successful in the sustained chemoprevention and treatment of the pts with Gorlin syndrome [15, 16]. Unfortunately, in the lack of concordance with monitoring skin lesions chemoprevention was not useful in this patient.

In the literature, there were limited data about optimal management of Gorlin syndrome. In addition to surgical excision and topical 5-fluorouracil (5-FU), systemic therapy modalities were reported to be infusional 5-FU, paclitaxel, and capecitabine therapies [17–19]. Our case underwent two surgical interventions and one session of local radiotherapy after the time of diagnosis. However, at his last admission, he was not found to be eligible for the surgery due to diffuse metastases and locally advanced primary tumor and therefore he began to receive systemic cisplatin and infusional 5-FU chemotherapy regimen.

Dysregulation in the hedgehog signaling pathway is the most important molecular abnormality underlying BCC. Vismodegib, an oral hedgehog signaling inhibitor, was reported to yield a response rate of 43% for locally advanced disease and 30% for metastatic BCC [20]. Vismodegib was shown to reduce the tumor burden in the basal cell nevus syndrome and to prevent the development of new lesions [21].

Consequently, in the patients with basal cell carcinoma with a low potential for metastasis development in the practice, a great emphasis should be placed on clinical monitoring and the patients should be informed about the eventual new lesions. It should be noted that the concomitance with any syndrome or the presence of multiple lesions with common recurrences may increase the risk for systemic metastasis and, in these patients, curative excision and, if needed, radiotherapy should be administered and they should be closely monitored for metastases.
